# Adoption and acceptability of a perioperative nutrition program among total joint arthroplasty patients

**DOI:** 10.3389/frhs.2025.1555209

**Published:** 2025-08-20

**Authors:** Derek S. Yocum, Leila Hammond, Emma Housholder, Néma McGlynn, Sara Y. Oikawa, Claudia Kabele, Adam Cien, Jeffrey D. Yergler

**Affiliations:** ^1^ Independent Researcher, South Bend Orthopaedics, South Bend, IN, United States; ^2^ Enhanced Medical Nutrition, Toronto, ON, Canada

**Keywords:** perioperative nutrition, arthroplasty, acceptability, patient optimization, surgery, adoption (ideas)

## Abstract

**Introduction:**

Perioperative nutrition interventions improve postoperative outcomes after total joint arthroplasty (TJA). Despite this improvement, adoption and acceptability of nutrition interventions among orthopedic patients have never been assessed, even though they are key factors in patient adherence and practitioner buy-in. To address this knowledge gap, the adoption and acceptability of a 4-week perioperative nutrition program were explored.

**Methods:**

Patients who underwent TJA at a single orthopedic clinic in South Bend, Indiana, USA, were invited to participate. Eligible patients were informed of the nutrition program > 2 weeks prior to their scheduled surgery. Two weeks postoperatively, patients were administered a digital questionnaire that captured their demographic information, whether they purchased the nutrition program or not, reasons for non-participation, and acceptability of the nutrition program among those who participated based on the theoretical framework of acceptability.

**Results:**

A total of 341 patients were approached, of which 208 consented. There were no demographic differences between the participants who purchased the nutrition program (105) and those who did not (103). The majority of the participants who purchased the nutrition program (78.7%, *n* = 85) were satisfied with it, and 65.7% (*n* = 71) believed it improved their surgical recovery. The most common reason cited for non-participation was cost.

**Conclusion:**

A nutrition program, implemented using a pragmatic adjunctive model, was adopted by many of the patients who underwent arthroplasty, regardless of demographics. The nutrition program was generally well-accepted by those who participated in it. Considerations around perceived cost need to be addressed in future implementations of the program.

## 1 Introduction

Total joint arthroplasty (TJA) is a common surgical procedure that involves the replacement of a damaged joint with an artificial one. While the procedure is often effective in reducing pain and improving mobility and quality of life (1), it causes significant physiological stress on the body, which can lead to complications and prolonged recovery time (2). Importantly, surgeons are demonstrating increasing interest in perioperative strategies to attenuate the surgical stress response associated with this procedure and improve surgical outcomes (3).

Nutrition-based interventions are low-risk strategies that can improve surgical outcomes (4, 5). Recent studies suggest that amino acid supplementation in the perioperative period may benefit patients undergoing TJA by mitigating tissue catabolism and maintaining musculoskeletal function in the acute and intermediate period (1, 6–15). Amino acids also have anti-inflammatory properties, which in turn reduce pain following TJA (12, 16). Similarly, preoperative carbohydrate loading has been shown to offset surgical stress, resulting in reduced postoperative hyperglycemia, insulin resistance, discomfort, and pain (17–21). Combined, these nutrition interventions could lighten the burden on the healthcare system by reducing length of stay, risk of complications, and readmission rates, while accelerating discharge readiness and improving patient experience (17, 22).

While several investigations have explored the role of nutrition in improving arthroplasty outcomes, patient adoption and acceptability of these interventions have not been adequately studied, despite being key factors in determining practitioner administration and patient participation (23, 24). Patient acceptability may be particularly important for healthcare interventions that require intermediate-term, repeated input from patients, such as nutrition interventions. Patients are also more likely to follow treatment recommendations if an intervention is acceptable, which in turn improves the effectiveness of such interventions (23, 24).

To address this knowledge gap, the purpose of this study was to investigate the adoption and acceptability of a 4-week perioperative nutrition program, consisting of whey protein isolate and a preoperative carbohydrate drink, among patients who underwent TJA.

## 2 Methods

### 2.1 Study design

This prospective, cross-sectional, mixed-methods study administered a survey to patients who underwent TJA. The survey collected demographic data, nutrition program participation or non-participation (adoption), and patient-reported acceptability of the nutrition program among the patients who participated.

### 2.2 Study population, procedure, and recruitment

Patients who underwent elective total hip arthroplasty (THA) or total knee arthroplasty (TKA) for degenerative disease or trauma, at a single ambulatory surgery center in South Bend, Indiana [South Bend Orthopaedics (SBO)], were invited to participate in this study. Patients were excluded if they underwent emergency TKA or THA, were pregnant or lactating, had type 1 diabetes, had an allergy to any of the ingredients contained within the nutrition program, or did not have an email address. To be eligible for the study, patients had to be 18 years of age or older and scheduled for elective TKA or THA due to degenerative disease or trauma. All patients who underwent these surgeries, under coinvestigators Dr. Jeffrey Yergler, and Dr. Adam Cien, were informed of the study at their presurgical visit by the study's principal investigator. Those who were eligible reviewed the consent form with the study's research assistant. The patients reviewed the study protocol at their own pace, and informed consent was digitally obtained from all participants using PatientIQ. A sample size of 200 participants was calculated based on previous data examining nutrition intervention satisfaction in oncology patients, as data in orthopedics were not available, a 95% confidence level (*α* = 0.05), and a 5% margin of error. Independent Review Board (IRB) approval was obtained for this study from Solutions IRB (IRB #IORG0007116).

### 2.3 Nutrition program

At the time of surgery scheduling, the patients were informed of the commercially available perioperative nutrition program (Ortho Nutrition Bundle, Enhanced Medical Nutrition, Toronto, Canada). Purchase of the nutrition program was voluntary and not required for participation in the study. Patients who decided to purchase the program did so via e-commerce or by phone directly from the manufacturer for $150 USD.

The nutrition program consisted of 56 servings of a whey protein isolate powder available in one of two flavors—vanilla or decaf cold brew (ISOlution®, Enhanced Medical Nutrition, Toronto, Canada) and two servings of a preoperative complex carbohydrate powder (PREcovery®, Enhanced Medical Nutrition, Toronto, Canada). The whey protein isolate provided 26g of protein and 3.6g of leucine per serving. The preoperative carbohydrate drink provided 50g of carbohydrate (maltodextrin) per serving. The nutrition program instructed patients to take one serving of the whey protein isolate twice daily between meals for 14 days preoperatively and 14 days postoperatively. The program also contained two servings of preoperative carbohydrate powder, each mixed into 400 ml of water, to be consumed the night before surgery. Adherence to the nutrition program was not assessed in this study.

### 2.4 Questionnaire

The questionnaire (Supplementary Materials S1) was developed in collaboration with dietitians and orthopedic surgeons. The questionnaire was adapted from the theoretical framework of acceptability (TFA), which assesses the acceptability of healthcare interventions in seven domains (25, 26). The domains of the TFA are as follows: affective attitude (how an individual feels about the intervention), burden (the perceived amount of effort that is required to participate in the intervention), perceived effectiveness (the extent to which the intervention is perceived as likely to achieve its purpose), ethicality (the extent to which the intervention has good fit with an individual's value system), intervention coherence (the extent to which the participant understands the intervention and how it works), opportunity costs (the extent to which benefits, profits, or values must be given up to engage in the intervention), and self-efficacy [the participant's confidence that they can perform the behavior(s) required to participate in the intervention] (25, 26).

The questionnaire contained four sections: (1) patient demographics, (2) surgical satisfaction, (3) nutrition program participation or non-participation, and (4) acceptability of the nutrition program. There were two versions of the questionnaire. One version was administered to the patients who participated in the nutrition program and consisted of 14 questions. The second version was administered to the patients who did not participate in the nutrition program and consisted of 10 questions.

While the questionnaire development was guided by the TFA validated by Sekhon et al. (25, 26), not all domains were captured through direct, single-question items. Specifically, opportunity cost was addressed indirectly through patient-reported reasons for non-participation and the perceived benefit of the program. In addition, given the clinical nature of the intervention and the lack of ethical or values-based objections reported in previous perioperative nutrition research (1, 5, 13), it was not anticipated that ethicality would be a primary determinant of acceptability in this population and thus this domain was also omitted from the questionnaire in the current study to reduce the number of questions and thus the burden on patients (1, 5, 13).

In addition, three contextual questions were included in the questionnaire that were not part of the original TFA. These items were included to provide important contextual data and support the interpretation of the acceptability findings. The additional questions and their justifications were as follows: “Is this your first joint replacement surgery?,” included to assess whether prior surgical experience influenced how patients perceived the value or novelty of the nutrition program; “How satisfied are you with your overall surgical experience?,” included to understand patients' baseline satisfaction with their surgery, independent of the nutrition program; “How do you think your recovery would be different if you participated in the nutrition program (Orthopedic Nutrition Bundle)?,” included to explore non-participants' perceived effectiveness and perceived opportunity cost. This question aimed to capture whether beliefs about recovery benefits influenced their decision not to participate.

### 2.5 Questionnaire administration

The survey was administered digitally to patients between June 2022 and April 2023 via PatientIQ on the patients' own devices or on a tablet provided to patients during their appointments. PatientIQ is a secure web-based platform and digital patient care pathway software that encompasses patient engagement and patient outcome data collection. Patients received email and text notifications to complete the questionnaire 14 days after their surgery through the PatientIQ platform. For those who had not completed the questionnaire prior to their 2-week postsurgical follow-up appointment at SBO, the survey was also made available in person via a tablet at that visit. To increase survey completion, a reminder was delivered to patients via PatientIQ a week after initial questionnaire deployment.

### 2.6 Statistical analysis

Statistical analyses were conducted using the statistical analysis tool in PatientIQ. A two-tailed *p*-value of ≤0.05 was considered statistically significant. Continuous variables are presented as mean ± SD and compared using the independent sample *t*-test or ANOVA when normal distribution was satisfied. In cases where normal distribution was not satisfied, continuous variables were compared using the non-parametric Mann–Whitney *U*-test. Ordinal categorical variables are expressed as frequency counts (percentage of total).

## 3 Results

### 3.1 Adoption and demographics

In total, 208 patients consented to join this study, of which 105 opted to participate in the nutrition program while 103 did not. The average age of the patients was 65 years. Most patients were white (82.7%, *n* = 172), married (68.8%, *n* = 143), and had attained a high school diploma (28.8%, *n* = 59) or at least some or more college education (70.2%, *n* = 143). Overall, patient demographics were similar between groups, with no differences between groups for gender (*p* = 0.481), ethnicity (*p* = 0.636), marital status (*p* = 0.979), age (*p* = 0.434), or education (*p* = 0.539) (Table 1).

**Table 1:** Participant demographics.

	Participants	Non-participants	*p*-Value
Patients (*N*)	105	103	
Gender			0.481
Female	53 (50.5%)	58 (56.3%)	
Male	52 (49.5%)	45 (43.7%)	
Ethnicity			0.636
Black or African American	4 (3.8%)	2 (1.9%)	
Unknown	16 (15.2%)	13 (12.6%)	
White	85 (81.0%)	87 (84.5%)	
Other race	0	1 (1.0%)	
Marital status			0.979
Divorced	6 (5.7%)	7 (6.8%)	
Domestic partner	1 (1.0%)	1 (1.0%)	
Married	71 (67.6%)	72 (69.9%)	
Single	19 (18.1%)	15 (14.6%)	
Unknown	1 (1.0%)	1 (1.0%)	
Widowed	7 (6.7%)	6 (5.8%)	
Separated	0	1 (1.0%)	
Age	65.362 ± 8.989	64.583 ± 8.650	0.434
What is your highest level of educational attainment?			0.539
9th–11th grade	3 (2.8%)	3 (2.9%)	
High school graduate	26 (24.1%)	33 (32.7%)	
Some college, no degree	26 (24.1%)	30 (28.8%)	
Associate's degree	10 (10.2%)	11 (10.6%)	
Bachelor's degree	21 (20.4%)	14 (13.5%)	
Master's degree	15 (14.8%)	9 (8.7%)	
Professional degree	3 (2.8%)	3 (2.9%)	
Doctoral degree	1 (0.9%)	0	
What is your marital status?			0.899
Never married	5 (4.6%)	7 (6.7%)	
Married	72 (68.5%)	72 (70.2%)	
Separated	1 (0.9%)	1 (1.0%)	
Divorced	18 (17.6%)	14 (13.5%)	
Widowed	9 (8.3%)	9 (8.7%)	
Is this your first joint replacement surgery?			0.109
Yes	57 (54.6%)	68 (66.3%)	
No	48 (45.4%)	35 (33.7%)	
How satisfied are you with your overall surgical experience?			0.0893
Very satisfied	65 (62.0%)	54 (52.4%)	
Satisfied	33 (31.5%)	36 (35.0%)	
Neutral	5 (4.6%)	13 (12.6%)	
Dissatisfied	1 (0.9%)	0	
Very dissatisfied	1 (0.9%)	0	

Continuous variables are presented as mean ± SD and were compared using the independent sample *t*-test or ANOVA when normal distribution was satisfied, otherwise it was expressed as median and quartile range and the difference between groups was compared using non-parametric tests (Mann–Whitney *U*-test). Ordinal categorical variables are expressed as frequency counts (percentage of total) and were compared using the Wilcoxon rank-sum test or Kruskal–Wallis *H*-test. A two-tailed *p-*value < 0.05 was considered statistically significant.

### 3.2 Number of joint replacements and surgery satisfaction

When comparing the nutrition program participant and non-participant group responses, there was a statistically equivalent number of nutrition program participants whose surgery was their first joint replacement (54.6%, *n* = 57) compared to the non-participants (66.3%, *n* = 68) (*p* = 0.11, Table 1). Surgical satisfaction was also statistically equivalent between the groups, as 93.5% (*n* = 98) of nutrition program participants and 87.4% (*n* = 90) of non-participants were “satisfied” or “very satisfied” with their surgical experience (*p* = 0.09, Table 1).

### 3.3 Impact of the nutrition program

Regarding the group-specific questions, the majority of the non-participants believed that their recovery would not have been different if they had participated in the nutrition program (89.4%, *n* = 92), with the remaining 10.6% (*n* = 11) believing that their surgical experience would have been “better” or “much better.” None of the non-participants thought their surgical experience would have been “worse” or “much worse.” The most common reason given for non-participation was that “it was too expensive” (38.8%, *n* = 40). Other reasons provided for not participating were that they did not believe they would benefit from it (13.6%, *n* = 14), they did not want to take nutrition supplements (7.8%, *n* = 8), they did not feel confident that they could participate in the nutrition program as required (3.9%, *n* = 4), they did not feel they had the time to participate in the program or that it was going to be too much effort (3.9%, *n* = 4), they did not think they would be satisfied with the nutrition program (1.9%, *n* = 2), they did not understand it 0.9% (*n* = 1), and 14.6% (*n* = 15) reported “other” as their reason for non-participation (Supplementary Materials S2). None of the participants reported that they did not understand the nutrition program as a reason for non-participation.

Among the nutrition program participants, the majority reported being “very satisfied” (40.7%, *n* = 43) or “satisfied” (38%, *n* = 40) with the nutrition program, and 88.9% (*n* = 96) either “agreed” or “strongly agreed” that they understood the program. Most of the nutrition program participants found the effort required to participate was “not much” (39.8%, *n* = 43) or “very little” (23.1%, *n* = 25), and the patients reported confidence in participating (45.4% “strongly agree,” *n* = 49 and 37% “agree,” *n* = 40). Overall, 65.7% (*n* = 71) of the nutrition program participants “agreed” or “strongly agreed” that the program was effective in improving their surgical recovery (Table 2).

**Table 2:** Nutrition program acceptability.

How satisfied are you with the nutrition program (Ortho Nutrition Bundle)? (measuring affective attitude)	
Very satisfied	43 (40.7%)
Satisfied	40 (38.0%)
Neutral	19 (18.5%)
Dissatisfied	1 (0.9%)
Very dissatisfied	2 (1.9%)
I understand how the nutrition program (Ortho Nutrition Bundle) works. (measuring intervention coherence)	
Strongly agree	28 (25.9%)
Agree	68 (63.0%)
Neutral	12 (11.1%)
How much effort was required of you to participate in the nutrition program (Ortho Nutrition Bundle)? (measuring burden)	
A great deal	4 (3.7%)
Much	4 (3.7%)
Some	32 (29.6%)
Not much	43 (39.8%)
Very little	25 (23.1%)
I am confident I participated in the nutrition program (Ortho Nutrition Bundle) as required. (measuring self-efficacy)	
Strongly agree	49 (45.4%)
Agree	40 (37.0%)
Neutral	14 (13.0%)
Disagree	1 (0.9%)
Strongly disagree	4 (3.7%)
I believe the nutrition program (Ortho Nutrition Bundle) was effective in improving my recovery after surgery. (measuring perceived effectiveness)	
Strongly agree	23 (21.3%)
Agree	48 (44.4%)
Neutral	36 (33.3%)
Strongly disagree	1 (0.9%)

Continuous variables are presented as mean ± SD and were compared using the independent sample *t*-test or ANOVA when normal distribution was satisfied, otherwise they are expressed as median and quartile range and the difference between groups was compared using non-parametric tests (Mann–Whitney *U*-test). Ordinal categorical variables are expressed as frequency counts (percentage of total) and were compared using the Wilcoxon rank**-**sum test or Kruskal–Wallis *H*-test. A two-tailed *p-*value < 0.05 was considered statistically significant.

## 4 Discussion

This study is the first to evaluate the adoption and acceptability of a 4-week perioperative nutrition program among patients who underwent arthroplasty. We found that half of the study participants purchased the nutrition program (51%, *n* = 105), and interestingly, the patient demographics were similar in the nutrition program participant and non-participant groups. This finding suggests that the adoption of the intervention was consistent regardless of the demographic factors evaluated. It also suggests that the nutrition program has the potential to be implemented and utilized across various patient populations without concerns of differential acceptability based on demographic factors. These findings support the idea that all patients, irrespective of clinic demographics, should be offered the opportunity to participate in a perioperative nutrition program. At present, however, perioperative nutrition is not part of the standard of care in most orthopedic practices (27).

Among those who participated in the nutrition program, acceptability was high. The participants in the nutrition program generally had a positive affective attitude toward the nutrition program as indicated by the high satisfaction ratings (78.7% “very satisfied” or “satisfied,” *n* = 83). The nutrition program participants also indicated that the burden of participation was low (62.9%, *n* = 68, reported it to be “not much” or “very little”) and that their self-efficacy for completing the program was high, with 82.4% (*n* = 89) indicating confidence that they participated in the nutrition program as required. Even though a framework regarding the effect of nutritional supplementation knowledge and self-efficacy on surgery outcomes has not been well studied, these components are important influencers on adherence to a nutritional intervention. In addition, it has been shown that a patient's self-efficacy toward their rehabilitation predicts recovery from orthopedic surgery (28).

In the current study, most of the nutrition program participants (65.7%, *n* = 71) felt that using the perioperative nutrition program was effective in improving their recovery after surgery, while many of the non-participants felt that their recovery would have been “the same” whether or not they had participated in the nutrition program (89.4%, *n* = 93). These data are in contrast to recent findings by Germano et al., who found that 89.2% (*n* = 267) of surveyed orthopedic surgery patients believed that nutritional supplementation would benefit surgical outcomes, suggesting a disconnect between nutrition beliefs and purchase intent (29). Germano et al. also reported that 84% (*n* = 252) of patients would purchase a nutrition program if it were recommended by their surgeon (29). Despite this finding, only half of the participants in the current study purchased the nutrition program as recommended by their surgeon. Interestingly, among all the participants in the study, the reported nutrition program intervention coherence, i.e., the extent to which the patient understood how the nutrition program worked, was high. None of the nutrition program participants indicated that they did not understand how the program worked, and none of the non-participants reported that a lack of understanding was the reason for not participating in the program, suggesting that the nutrition program and its associated use were not a barrier to participation. Future research should aim to elucidate the disconnect between nutrition beliefs and knowledge and purchase intent regarding perioperative nutrition supplements.

Considering that the patients' nutrition status and knowledge of nutrition optimization for surgery were not assessed in this study, evaluating these factors could help determine more effective ways for surgeons to educate patients on the usefulness of nutrition interventions for TJA and thus improve their adoption in this surgical population (30, 31).

While there were no differences in demographics between the nutrition program participants and non-participants, the cost of the nutrition program was the leading reason for non-participation. Unfortunately, demographic data associated with total household income was not collected. As a result, we cannot conclude that socioeconomic status was the determining factor for non-participation in the nutrition program, and this limited our ability to assess the socioeconomic drivers of uptake. Considering the equivalence between the two groups for all the other demographic domains collected in the survey, it is possible that household income was not different between the groups. If true, this finding suggests that the perceived financial cost of the nutrition program was a barrier for the majority of non-participants. Regardless, the financial implications of such interventions should be carefully considered when assessing their acceptability and planning for implementation. The affordability of healthcare interventions is a critical consideration in the adoption of an intervention, as it impacts patient access and adherence (32). At $150 USD, the cost of the nutrition program was a barrier for many patients, which raises concerns about equity and the potential for disparities in access to the intervention based on socioeconomic status. Policy solutions and subsidization efforts are critical to ensure patients have access to evidence-based nutrition products to support optimal surgical recovery. Given their potential to improve outcomes and reduce complications, hospitals and insurance networks should consider integrating these products into the standard of perioperative care.

### 4.1 Study strengths

The major strength of this study is its ecological relevance. It evaluated the adoption and acceptability of the nutrition program in a model consistent with how it is implemented in clinical settings. This study was also the first to assess patient-reported acceptability of a perioperative nutrition program in the context of arthroplasty. Previous research on nutrition interventions among this patient population has been limited to randomized controlled trials that did not provide information on the patient adoption and acceptability of these kinds of nutrition interventions (1, 6, 8–14, 33, 34). This study provided the much-needed patient perspective feasibility data required for the implementation of these kinds of interventions. Furthermore, the use of a patient engagement platform (PatientIQ) for data collection provided an unintrusive and efficient way to collect survey responses.

### 4.2 Study limitations and future research

This study was, to our knowledge, the first to explore the acceptability of a nutrition program using a TFA. However, we acknowledge several limitations that limit the applicability of our findings. Importantly, two TFA constructs, namely opportunity costs and ethicality, were not explicitly represented as stand-alone items in the current questionnaire. Instead, opportunity cost was addressed indirectly through patient-reported reasons for non-participation and the perceived benefit of the program. Ethicality was not measured due to the clinical nature of the intervention and the absence of value conflicts in prior studies. Future research should incorporate these domains more explicitly as the TFA is further refined and adapted for perioperative nutrition interventions in surgical populations. In addition, although the administered questionnaire was based on a validated framework, it had not been tested for validity among an orthopedic population prior to deployment (26). Employing methods to ensure questionnaire reliability and validity will ensure it is psychometrically sound, and these should be conducted prior to replicating further research on the acceptability of nutrition interventions among orthopedic patients. In conjunction with a lack of validity is a lack of generalizability. This study assessed patient adoption, demographics, and acceptability among patients who underwent TJA from only two surgeons at a single orthopedic clinic, limiting the generalizability of these findings. Future studies evaluating this intervention in patients undergoing TJA at other orthopedic clinics are necessary. Furthermore, this study did not assess household income. As a result, it is unknown whether household income was higher among the nutrition program participants compared to non-participants. Alternatively, if household income was similar, the perceived financial cost of the nutrition program was a potential barrier. This uncertainty limits our understanding of how this variable may have influenced the adoption of the nutrition program, especially since cost was the most cited reason for non-participation. Future studies should include household income as a demographic variable.

## 5 Conclusion

This study shows that of the participants in this study offered a perioperative nutrition program for TJA at SBO, half adopted it. Patient acceptability of the nutrition program, among those who participated in it, was high, with the majority reporting they felt satisfied with it, understood how it worked, felt it had a low participation burden, felt confident that they participated as required, and believed it had a positive effect on their surgical recovery. Despite the positive relationship found between nutrition program participation and acceptability, there was an equal number of patients who did not participate in the nutrition program, with cost being the main reason. The findings of this study can be used to implement nutrition programs for patients undergoing arthroplasty surgery in the United States. More research is required to ensure questionnaire validity and investigate factors impacting financial barriers to nutrition program adoption.

## Data Availability

The raw data supporting the conclusions of this article will be made available by the authors, without undue reservation.
