# Supplementary material for: Adoption and acceptability of a perioperative nutrition program among total joint arthroplasty patients
Source: Front Health Serv. 2025 Aug 20;5:1555209. doi: 10.3389/frhs.2025.1555209 (PMC12405422; doi:10.3389/frhs.2025.1555209)
Supplement: Supplementary file 1 [file Datasheet1.pdf]

SOUTH BEND ORTHOPAEDICS
A DIVISION OF ASCENDANT ORTHOPEDIC ALLIANCE, LLC

We would like to gather your feedback on a nutrition program (Ortho Nutrition Bundle), that was presented to you at your consultation visit. The survey will take about 10 mins to complete and begins with a few demographic questions. Thank you for your participation.

Age? (Will be retrieved from chart in years)

1. Sex assigned at birth?
   - Male
   - Female
   - Intersex
   - Prefer not to say

2. What is your highest level of educational attainment?
   - None- 8th grade
   - 9th-11th grade
   - High school graduate
   - Some college, no degree
   - Associate's degree
   - Bachelor's degree
   - Master's degree
   - Professional degree
   - Doctoral degree

3. What is your ethnicity (select all that apply)?
   - White
   - Black/ African American
   - American Indian/Alaska Native
   - Asian
   - Native Hawaiin/ Other Pacific Islander
   - Hispanic/Latino

4. What is your marital status?
   - Never Married
   - Married
   - Separated
   - Divorced
   - Widowed

5. Is this your first joint replacement surgery?
   - Yes
   - No

6. How satisfied are you with your overall surgical experience?

- Very satisfied
- Satisfied
- Neutral
- Dissatisfied
- Very Dissatisfied

7. Did you participate in the nutrition program (Ortho Nutrition Bundle)?
   - Yes (Proceed to Question 10 and rest of survey)
   - No (Proceed to Question 8, followed by question 9 (to help determine potential for these participants to use the ONB in upcoming surgeries), then question 16)

8. Why did you not participate in the nutrition program (Orthopedic Nutrition Bundle). Select all that apply.
   - I did not feel I would be satisfied with the program
   - I did not understand it
   - I did not believe I would benefit from it
   - It was too expensive
   - I did not want to take nutrition supplements
   - I did not feel confident that I could participate in the program as required
   - I did not have the time to participate/it was going to be too much effort
   - Other___________

9. How do you think your recovery would be different if you participated in the nutrition program (Orthopedic Nutrition Bundle)?
   - Much Better
   - Better
   - The Same
   - Worse
   - Much Worse

10. How satisfied are you with the nutrition program (Ortho Nutrition Bundle)?
    - Very satisfied
    - Satisfied
    - Neutral
    - Dissatisfied
    - Very Dissatisfied

11. I understand how the nutrition program (Ortho Nutrition Bundle) works?
    - Strongly Agree
    - Agree
    - Neutral
    - Disagree
    - Strongly Disagree

SOUTH BEND ORTHOPAEDICS
A DIVISION OF ASCENDANT ORTHOPEDIC ALLIANCE, LLC

12. How much effort was required of you to participate in the nutrition program (Ortho Nutrition Bundle)?
    - A Great Deal
    - Much
    - Some
    - Not Much
    - Very Little

13. I am confident I participated in the nutrition program (Ortho Nutrition Bundle) as required?
    - Strongly Agree
    - Agree
    - Neutral
    - Disagree
    - Strongly Disagree

14. How likely is it that you would recommend the nutrition program (Ortho Nutrition Bundle) to a friend or colleague?

0	1	2	3	4	5	6	7	8	9	10

Not at all likely
Extremely likely

15. I believe the nutrition program (Ortho Nutrition Bundle) was effective for improving my recovery after surgery?
    - Strongly agree
    - Agree
    - Neutral
    - Disagree
    - Strongly Disagree

16. Please provide any other feedback you have about the nutrition program (Ortho Nutrition Bundle) and/or South Bend Orthopaedics.

____
____
____
____
